# Prevalence of sleep disorders among medical students and their association with poor academic performance: A cross-sectional study

**DOI:** 10.1016/j.amsu.2020.08.046

**Published:** 2020-09-08

**Authors:** Ahmed Yassin, Abdel-Hameed Al-Mistarehi, Othman Beni Yonis, Abdelwahab J. Aleshawi, Suleiman M. Momany, Basheer Y. Khassawneh

**Affiliations:** a Division of Neurology, Department of Neuroscience, Faculty of Medicine, Jordan University of Science and Technology, Irbid, Jordan; b Department of Public Health and Family Medicine, Faculty of Medicine, Jordan University of Science and Technology, Irbid, Jordan; c Department of Ophthalmology, Faculty of Medicine, Jordan University of Science and Technology, Irbid, Jordan; d Division of Pulmonary, Critical Care & Sleep Disorders, Department of Internal Medicine, Faculty of Medicine, Jordan University of Science and Technology, Irbid, Jordan

**Keywords:** Sleep disorders, Medical students, Academic performance, Epidemiology, Sleep apnea, Insomnia

## Abstract

**Background:**

Sleep quality is of paramount importance for human health. This multi-site study measures the proportion and types of self-reported sleep disorders in medical students and evaluates their association with academic performance by Grade Point Average (GPA).

**Materials and methods:**

A cross-sectional survey was conducted on medical students from two medical schools in Jordan during the 2018/2019 academic year. The study utilized the SLEEP-50 questionnaire to estimate the proportion of several sleep disorders and their effects on daily functioning. Below average GPAs were considered poor academic performance.

**Results:**

1041 medical students' online surveys were analyzed from two medical schools’ campuses, representing a 29.7% response rate. Their mean age was 22 ± 2.1 years (ranging from 18 to 37) and 52.6% were female. The mean body mass index was 24.2 ± 4.4 kg/m^2^. According to the SLEEP-50 questionnaire, the prevalence of sleep disorders among studied medical students ranged from 0.6% for sleep state misperception (SSM) to 23.1% for hypersomnia. Using binary logistic regression, after adjusting for gender and obesity, poor academic performance was associated with a risk for insomnia [adjusted odds ratio (OR) = 1.96, p < 0.001]; affective disorder [OR = 2.24, P < 0.001]; SSM [OR = 6.40, p = 0.045]; narcolepsy [OR = 9.54, p = 0.045]; and circadian rhythm disorders [OR = 2.03, p < 0.001].

**Conclusion:**

Sleep disorders are common among medical students. Several sleep

disorders were associated with poor academic performance. Proper diagnosis and treatment of sleep disorders may remedy this issue.

## Highlights

• Sleep disorders such as hypersomnia and insomnia are common among medical students.
• Several sleep disorders have a negative association with their academic performance.
• Insomnia, affective disorder, SSM, narcolepsy, and CRD are risk factors for poor academic performance.
• Proper diagnosis and treatment of sleep disorders may improve the academic performance.

## 1 Introduction

Adequate sleep is important for human's mental and physical well-being and chronic sleep deprivation has been linked to impaired neurobehavioral functioning [1]. The prevalence of sleep disorders varies but reportedly affects 22%–65% of the general population [[2], [3], [4]]. Approximately, one-third of adults report some form of insomnia [5,6]. Obstructive sleep apnea (OSA) is found in approximately 16%–36% of the general population and is usually associated with insomnia or excessive sleepiness [[7], [8], [9]]. There is growing evidence on the presence of risk factors and symptoms of several sleep disorders among college students [10]. Several studies have found a relatively high prevalence of sleep-related complaints, e.g. inadequate sleep, difficulty falling asleep or maintaining sleep, early morning awakenings, poor sleep quality, early morning sleepiness, and daytime napping, among college students [[11], [12], [13], [14], [15]]. Previous studies also showed the detrimental impact of several sleep disorders, snoring, and daytime sleepiness on the academic performance of college students [[16], [17], [18], [19], [20], [21]].

Among college students, medical students are under particularly high levels of stress, hence the crucial need for adequate refreshing sleep (to maintain cognitive and physical well-being) to achieve their goals [22]. A previous study showed a high prevalence of symptoms and an elevated risk of several sleep disorders among medical students [12]. Our study sought to find out the proportion of different sleep disorders among medical students and to determine their association with academic performance.

## 2 Participants and methods

### 2.1 Study participants

Jordan is a small Middle Eastern country with six medical schools. The Doctor of Medicine curriculum in Jordanian universities is a six-year degree designed to provide undergraduates with basic biomedical sciences and clinical skills training. The present study is a cross-sectional survey that was conducted during the 2018/2019 academic year at two academic medical schools in the north of Jordan: Jordan University of Science and Technology (JUST) and Yarmouk University (YU). 2517 and 984 undergraduate medical students were the total number of registered medical students at JUST and YU, respectively. After obtaining institutional review board approval, the questionnaire was sent to all registered medical students by email. Participation was voluntary and students were informed their responses would be confidential. The students did not receive any incentives or rewards for their participation in the study. The questionnaire was distributed in its original version in the English language, the official teaching language in medical schools of Jordanian universities.

### 2.2 Study questionnaire and data collection

The validated SLEEP-50 questionnaire by Spoormaker et al. [23] was used in this study. The questionnaire consists of 50 questions which screen for 10 sleep disorders including OSA, insomnia, affective disorder, sleep state misperception (SSM), narcolepsy, restless leg syndrome/periodic limb movement disorder (RLS/PLMD), circadian rhythm disorder (CRD), sleepwalking, nightmares, and hypersomnia. The internal consistency of the SLEEP-50 scale is 0.85 and its test-retest reliability falls between 0.65 and 0.89 [23]. The SLEEP-50 has good sensitivity and specificity and the agreement between all clinical diagnoses and SLEEP-50-classifications is substantial (κ = 0.77) [23]. The questionnaire consists of nine sections, each with three to eight items inquiring about different sleep-related complaints. The response to each item (e.g. I am told that I snore) is rated as 1 (not at all), 2 (somewhat), 3 (rather much), and 4 (very much) based on the student's sleep quality in the four weeks prior to the completion of the questionnaire. A minimum score of three or four is necessary for a response to be significant, i.e. be considered a sleep complaint. Each sleep disorder requires at least one sleep complaint and a minimum sub-score to be defined. The latter is calculated by adding the scores of its related items (e.g for defining OSA, there are eight items with a score of 1–4 for each item. A total sub-score should be ≥ 15 and one or more of these items should be rated 3 or 4 to define OSA) **(**Table 1)**.** An additional set of questions was used to collect university identification numbers (IDs), sociodemographic data, and students' sleep habits. Students were considered short, normal, and long sleepers based on whether they slept less than six, six to eight, or more than 8 hours a day, respectively [24]. In July of 2019, Grade Point Averages (GPAs) for the 2018/2019 academic year were provided by the registration office of each medical school. To protect confidentiality, the corresponding author was the only person who had access to GPAs. Academic grades are classified as below average (GPA ≤ 2.49), good (GPA 2.50–2.99), very good (GPA 3.00–3.49), excellent (GPA 3.50–3.99), and outstanding (GPA ≥ 4.00). Student’ academic performance was defined as poor if their GPA was below average.

**Table 1:** Sleep disorders based on the SLEEP-50 questionnaire

Disorder	Items	Subscore
Obstructive Sleep Apnea	1–8	≥15
Insomnia	9–16	≥19
Affective disorder	10, 11, 43, 44	≥12
Sleep state misperception	Insomnia, estimated hours slept <4	≥19
Narcolepsy	17–21	≥7
Restless legs syndrome/Periodic limb movement disorder	22–25	≥7
Circadian rhythm disorder	26–28	≥8
Sleep walking	29–31	≥7
Nightmares	32 33–35	≥3 ≥9
Hypersomnia	44–50	No item or ≥15 on impact of sleep disorder on daily functioning
All sleep disorders		≥15 on impact of sleep disorder on daily functioning

Informed consent was obtained from the recruited participants. They were not involved in the design of this study, or the study steering committee, and they were not provided inputs on outcomes selection. All procedures performed in this study involving human participants were reviewed and ethically approved by the Institutional Review Board (IRB) and research and ethics committee at JUST. This study was conducted following the 1975 Helsinki declaration (including its later amendments). This work has been reported based on STROCSS 2019 guidelines, and research protocol was registered in the Research Registry with the unique identification number of 5841 [25].

### 2.3 Statistical analysis

The characteristics of participants were described using frequency and percentage for categorical variables and mean ± standard deviation for continuous variables. A chi-square test was used to assess the association between categorical variables. A binary logistic regression analysis was performed to identify the association between each sleep disorder and poor academic performance while adjusting for gender and obesity. Odds ratios (OR) and their 95% confidence intervals (95% CI) were reported. A p-value of less than 0.05 was considered statistically significant. Version 25.0 of the Statistical Package for Social Sciences Software was used for data analysis.

## 3 Results

**Fig. 1:**
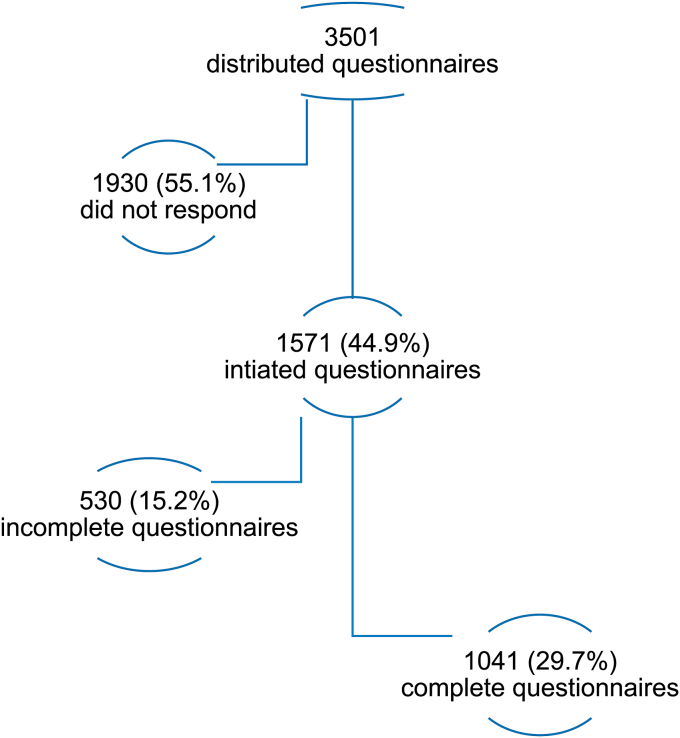
Study participants.

A total of 1571 students (44.9%) completed the online questionnaire. After data revision, 530 were excluded due to missing information or invalid university IDs. Data analysis was done on 1041 undergraduate medical students (29.7%). Their mean age was 22 ± 2.1 years (ranging from 18 to 37), and 52.6% were female (Fig. 1). The mean body mass index was 24.2 ± 4.4 kg/m^2^. The majority of students (713, 68.5%) were in their clinical (fourth to sixth) years (Table 2). More than one-quarter (320, 30.7%) of the students were short sleepers, more than half (565, 54.3%) were normal sleepers, and one-fifth (156, 15%) were long sleepers. Only 40 students (3.8%) were previously diagnosed with a sleep disorder: 28 had hypersomnia, 5 had OSA, 5 had insomnia, and 2 had RLS. Academic performance was outstanding/excellent in 18.5% of the students, very good in 39.1%, good in 23.5%, and below average in 18.8%. Poor academic performance was more frequent in male (P < 0.001) and obese students (P < 0.001).

**Table 2:** Demographic distribution of undergraduate medical students.

Total number of respondents = 1041	
**University**	
JUST	846 (81.3%)
Yarmouk University	195 (18.7%)
**Gender**	
Male	493 (47.4%)
Female	548 (52.6%)
Age (year) mean ± SD	22.1 ± 2.1
Weight (Kg) mean ± SD	70.0 ± 17.4
Height (cm) mean ± SD	169.2 ± 9.4
BMI Kg/m^2^ mean ± SD	24.2 ± 4.4
Obese (BMI ≥ 30 kg/m2)	98 (9.4%)
GPA mean ± SD	3.3 ± 0.5
**School Year**	
2	164 (15.8%)
3	164 (15.8%)
4	195 (18.7%)
5	238 (22.9%)
6	280 (26.9%)

JUST: Jordan University of Science and Technology

**Fig. 2:**
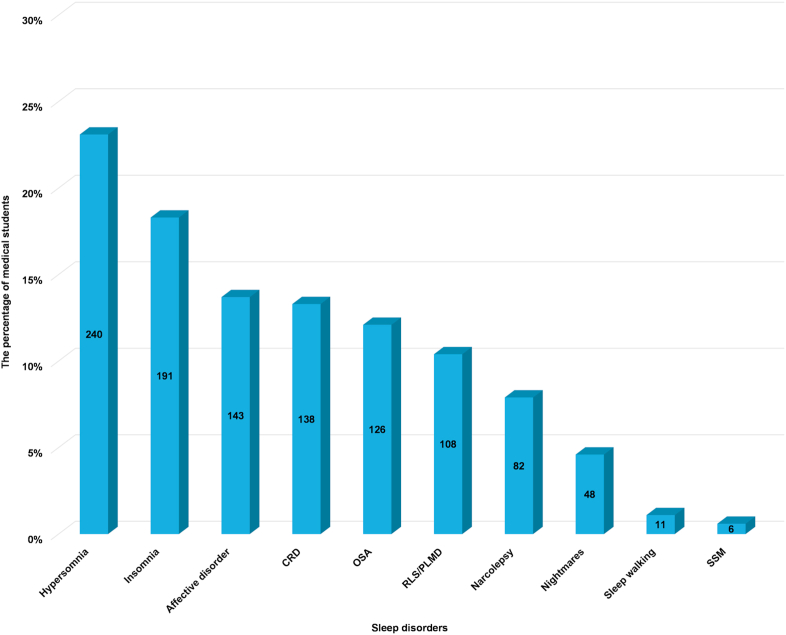
The proportion of sleep disorders among undergraduate medical students as ordered from the highest to lowest proportion

The use of the SLEEP-50 questionnaire showed that 689 students (66.2%) had at least one sleep disorder. Hypersomnia and insomnia were the most common sleep disorders (23.1% and 18.3%, respectively), followed by affective disorder (13.7%), CRD (13.3%), OSA (12.1%), RLS/PLMD (10.4%), narcolepsy (7.9%), nightmares (4.6%), sleepwalking (1.1%) and SSM (0.6%) (Fig. 2). Male medical students were at higher risk of OSA (76, 15.4%) compared with female students (50, 9.1%) with the unadjusted odds ratio (OR) of 1.815 (95% CI 1.242–2.654, p = 0.002). On the other hand, female students had a higher risk of RLS/PLMD (72, 13.1%) and nightmares (33, 6.0%) compared with male students (36, 7.3% and 15, 3.0%, respectively). The unadjusted OR for RLS/PLMD was 0.521 (95% CI = 0.342–0.793, p = 0.002), and 0.490 for nightmares (95% CI = 0.263–0.913, p = 0.022). There was no significant difference between males and females regarding insomnia (p = 0.694), affective disorder (p = 0.554), SSM (p = 0.897), narcolepsy (p = 0.337), CRD (p = 0.162), sleep walking (p = 0.090), or hypersomnia (p = 0.492) (Table 3).

**Table 3:** The proportion of sleep disorders among undergraduate medical students by the effect of gender

Sleep Disorder	Female n = 548	Male n = 493	p value (χ^2^)
OSA	50 (9.1%)	76 (15.4%)	0.002
Insomnia	103 (18.8%)	88 (17.8%)	0.694
Affective disorder	72 (13.1%)	71 (14.4%)	0.554
SSM	3 (0.5%)	3 (0.6%)	0.897
Narcolepsy	39 (7.1%)	43 (8.7%)	0.337
RLS/PLMD	72 (13.1%)	36 (7.3%)	0.002
CRD	65 (11.9%)	73 (14.8%)	0.162
Sleep walking	3 (0.5%)	8 (1.6%)	0.090
Nightmares	33 (6.0%)	15 (3.0%)	0.022
Hypersomnia	131 (23.9%)	109 (22.1%)	0.492

OSA: obstructive sleep apnea; SSM: sleep state misperception; RLS/PLMD: restless leg syndrome/periodic limb movement disorder; CRD: circadian rhythm disorder.

**Fig. 3:**
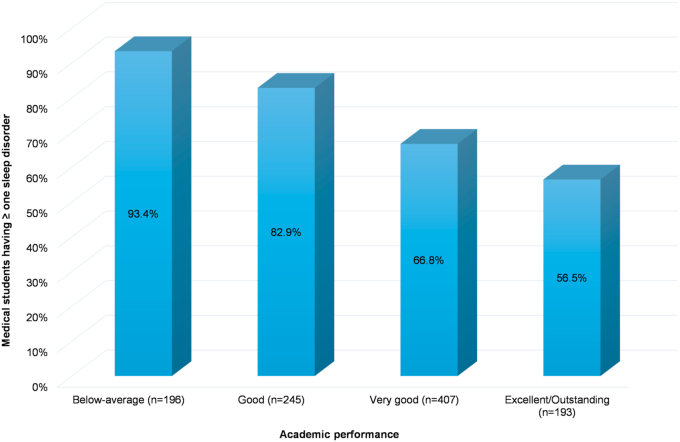
The percentage of medical students having ≥ one sleep disorder is inversely related to their academic performance.

Students with below average academic performance more commonly had at least one sleep disorder when compared to those with very good/excellent/outstanding performance (p = 0.005) (Fig. 3). Students at high risk of OSA, insomnia, affective disorder, SSM, narcolepsy or CRD were more likely to have poor academic performance compared to those at lower risk for these sleep disorders (P < 0.05) (Table 4).

**Table 4:** Students’ characteristics and the association of sleep disorders with academic performance

Variables, n (%)	Poor performance, GPA ≤ 2.49 (n = 196)	Good performance, GPA ≥ 2.50 (n = 845)	p value (χ2 test)
**Gender:**			<0.001
Female	81 (41.3%)	467 (55.3%)	
Male	115 (58.7%)	378 (44.7%)	
**Obesity** (BMI ≥ 30 kg/m^2^)	41 (20.9%)	57 (6.7%)	<0.001
**School Year:**			<0.001
Second Year	38 (19.4%)	126 (14.9%)	
Third Year	43 (21.9%)	121 (14.3%)	
Fourth Year	47 (24.0%)	148 (17.5%)	
Fifth Year	23 (11.7%)	215 (25.4%)	
Sixth Year	45 (23.0%)	235 (27.8%)	
**Academic level:**			0.001
Preclinical	81 (41.3%)	247 (29.2%)	
Clinical	115 (58.7%)	598 (70.8%)	
**Sleep Disorders:**			
OSA	33 (16.8%)	93 (11.0%)	0.024
Insomnia	53 (27.0%)	138 (16.3%)	<0.001
Affective disorder	45 (23.0%)	98 (11.6%)	<0.001
SSM	4 (2.0%)	2 (0.2%)	0.003
Narcolepsy	51 (26.0%)	31 (3.7%)	<0.001
RLS/PLMD	22 (11.2%)	86 (10.2%)	0.665
CRD	41 (20.9%)	97 (11.5%)	<0.001
Sleep walking	4 (2.0%)	7 (0.8%)	0.135
Nightmares	11 (5.6%)	37 (4.4%)	0.458
Hypersomnia	38 (19.4%)	202 (23.9%)	0.176

OSA: obstructive sleep apnea; SSM: sleep state misperception; RLS/PLMD: restless leg syndrome/periodic limb movement disorder; CRD: circadian rhythm disorder.

Using binary logistic regression while adjusting for gender and obesity, several sleep disorders were significantly associated with poor academic performance: insomnia (adjusted OR 1.96; 95% CI 1.35–2.85; p < 0.001), affective disorder (adjusted OR 2.24; 95% CI 1.50–3.35; p < 0.001), SSM (adjusted OR 6.40; 95% CI 1.04–39.19; p = 0.045), narcolepsy (adjusted OR 9.54; 95% CI 5.83–15.60; p = 0.045), and CRD (adjusted OR 2.03; 95% CI 1.34–3.08; p < 0.001). Interestingly OSA, when adjusted for gender and obesity, did not show a significant association with poor academic performance (p = 0.117), while male gender and obesity were significantly associated with poor academic performance (adjusted OR 1.44; 95% CI 1.03–2.00; p = 0.031) and (adjusted OR 3.13; 95% CI 1.99–4.91; p < 0.001), respectively.

## 4 Discussion

This study aimed to discover the proportion of sleep disorders among medical students and examine their association with academic performance through the use of a well-validated screening tool. Approximately two-thirds of medical students were at risk of at least one sleep disorder, and several sleep disorders had a negative impact on academic performance. In this study, hypersomnia was more prevalent in medical students (23.1%) than in the general adult population (3.9%–16%), as reported in previous community-based studies [26,27]. Insomnia was also prominent in this study (18.3% of the medical students). Gaultney et al. study, using SLEEP-50 questionnaire that surveyed 1845 students enrolled in Introductory Psychology labs at a large state university in the United States, showed that insomnia was one of the most reported disorders (12%) [16]. Piro et al. study, using the same questionnaire, conducted on a smaller cohort, 316 students from medical colleges (medicine, nursing, dentistry, pharmacy, anesthesia, and medical laboratory sciences) at the public University of Duhok-Iraq, found that RLS was the most prevalent type of sleep disorder, affecting 30.7% of students, followed by insomnia (25.0%), CRD (19.6%), affective disorder (14.5%), and sleep apnea (13.6%) [28]. This variation in the prevalence rate of different sleep disorders could be attributed to the different studied cohorts, and different methods and designs used in the studies.

In regard to gender, the results of this study are congruent with previous studies on the general population which showed that the prevalence of OSA in men was double that in women [[29], [30], [31]]. Piro et al. showed that women were at greater risk for RLS/PLMD and nightmares, but there was no gender difference in the risk of OSA [28].

The present study also found that the percentage of medical students with at least one sleep disorder progressively increased as the GPA decreased. Academic performance was negatively associated with the presence of high risk for OSA, insomnia, affective disorder, SSM, narcolepsy, and CRD. Using binary logistic regression analysis adjusting for gender and obesity, poor academic performance was nine times higher in those at high risk for narcolepsy, six times higher in those at high risk for SSM, and twice higher in those at high risk for insomnia, affective disorder, or CRD. Interestingly, after adjusting for the effects of gender and obesity, OSA was not associated with poor academic performance. This may be explained by the fact that both the male gender and obesity are known major risk factors for OSA [32,33]. Male as well as obese medical students in our study were at higher risk of having poor academic performance. Therefore, adjusting for these two confounding variables would make the association between OSA and poor academic performance insignificant. Gaultney et al. study on college students showed that academic failure was linked to the risk of OSA, insomnia, and CRD [16]. However, this study did not adjust for the effects of confounding factors such as gender and obesity. Piro et al. study showed that insomnia, affective disorder, and having multiple sleep disorders were associated with lower GPA [28]. The latter study was limited by a small sample size, 316 students, and a different target population.

It is plausible to assume that the daytime sleepiness, low attention levels and impaired memory/decision making that follow a lack of proper sleep are the explanation for poor academic performance in medical students with sleep disorders [34,35].

Our study also showed that a significant percentage of medical students were short sleepers (30.7%). A cross-sectional survey of approximately 50,000 Norwegian college and university students showed that both sleeping less than 5 h and more than 10 h were associated with failing exams compared with those sleeping seven to 9 h [36]. Short sleep duration and poor sleep quality among medical students could be ascribed to their huge academic load, professional attitudes and habits, and poor awareness of sleep hygiene [10]. The consequences of such behavior can be detrimental to cognitive and behavioral performances [1,37].

This study has a few limitations that should be mentioned. Approximately one-third of all registered medical students were included in the analysis. Although this may create a nonresponse bias, studies have shown that the average response rate of email-based surveys ranges between 25 and 30% [38]. There is high percentage of participants from one college in comparison to the other (846 (81%) from JUST versus 195 (19%) from YU); this could be attributed to the different population size in these colleges (2517 at JUST versus 984 at YU). Although SLEEP-50 is a validated screening tool for the most prevalent DSM–IV–TR sleep disorders (sleep apnea, insomnia, RLS, PLMD, CRD, and nightmares), its predictive validity for SSM, narcolepsy, and affective disorder is limited [23]. Moreover, SLEEP-50 is not a confirmatory diagnostic test for the evaluated sleep disorders. Further studies that utilize more definitive diagnostic tools (e.g., polysomnography) are needed to better investigate the association between sleep disorders and academic performance.

The findings of the study are invaluable for college administrators, healthcare promoters, and health counselors to draw their attention to common sleep problems among medical students. The results of this study encourage the medical schools to increase awareness of the negative impact of sleep disorders on academic performance. Despite the need for further research to establish a cause–effect association, the responsible professionals for students’ health can take action to improve the sleep patterns of medical students. It is worth to inform medical students of the consequences of bad sleep habits and sleep disorders through awareness programs, and encourage students to consult with sleep physicians to diagnose and manage any suspected sleep disorders.

## 5 Conclusion

Sleep disorders, particularly hypersomnia and insomnia, are common among undergraduate medical students. Several sleep disorders such as insomnia, affective disorder, SSM, narcolepsy, and CRD are associated with poor academic performance. Prospective studies that further compare the academic performance of medical students (before and after the diagnosis and treatment of sleep disorders) will be useful to clarify the association between sleep disorders and poor academic performance. Establishing such association can emphasize the importance of diagnosing and treating sleep disorders among medical students and consequently improve the outcome of medical schools.

## Funding

No Funding was received for this study.

## Declaration of competing interest

Authors declare that they have no conflicts of interest.
